# Research Letter: Antihypertensive Drugs Market in India: An Insight on Size, Trends, and Prescribing Preferences in the Private Health Sector, 2016–2018

**DOI:** 10.5334/gh.999

**Published:** 2021-08-02

**Authors:** Swagata Kumar Sahoo, Anupam Khungar Pathni, Ashish Krishna, Andrew E. Moran, Jennifer Cohn, Sanchit Bhatia, Nilesh Maheshwari, Bhawna Sharma

**Affiliations:** 1 Resolve to Save Lives, an initiative of Vital Strategies, New Delhi, IN; 2 Resolve to Save Lives, an initiative of Vital Strategies, New York, US; 3 Columbia University Irving Medical Center, New York, US; 4 Division of Infectious Diseases, University of Pennsylvania School of Medicine, Philadelphia, US; 5 Department of Analytics, IQVIA Consulting and Information Services, New Delhi, IN

**Keywords:** Antihypertensive drugs, single-pill combinations, drugs market, hypertension treatment

## Abstract

**Background:**

India has a high burden of hypertension. While the private sector provides 70% of out-patient care in the country, a significant proportion of patients seeking care from the public sector buy drugs from private markets. This study aimed to describe India’s private sector antihypertensive drugs market at the national and state levels over 2016–2018.

**Methods:**

Antihypertensive drugs sales in India from 2016–2018 were analysed using a large nationally representative dataset for the private pharmaceuticals market. In addition, data for five states (Punjab, Madhya Pradesh, Kerala, Telangana, and Maharashtra) that were the foci of a large hypertension control program were studied.

**Results:**

The Indian hypertension drug market grew at a rate of 6.9% from 2016 to 2018 with a total of 21,066 million pills sales in 2018. Single-pill combinations (SPCs) contributed to 39.1% of total sale volumes. The market comprised of 182 different antihypertensive drugs including 134 SPCs. Total volume of sales covered a maximum of 26% of treatment need for the estimated population with hypertension. Two-drug SPCs had the highest market share (36%), followed by calcium channel blockers (18%), beta-blockers (16%) and angiotensin receptor blockers (14%). Among SPCs, amlodipine+atenolol had highest sales (9.8%). Twenty-five drugs, a mix of single drugs and SPCs, accounted for 80% of total sales. There were large state-to-state variations in sales per capita, preferred therapeutic classes and drugs.

**Conclusions:**

Despite the large antihypertensive drugs market, there exists a high unmet need for treatment in India. Inter-state differences in product sales indicate variable treatment practices, underscoring the need for private sector engagement to improve hypertension care practices aligned with national and international guidelines. SPCs contributed to a large share of the private market and inclusion of select antihypertensive SPCs in the national list of essential medications should be considered for the public health system.

India has a high burden of hypertension. While the private sector provides 70% of outpatient care in the country [1], a significant proportion of patients seeking care from the public sector may also buy drugs from private markets. This study aimed to describe India’s private-sector antihypertensive drugs market at the national and state levels during 2016–2018.

Antihypertensive drugs sales in India from 2016–2018 were analysed using a large nationally representative dataset for the private pharmaceuticals market, collected by IQVIA, a private healthcare market research company. In addition, data for five states (Punjab, Madhya Pradesh, Kerala, Telangana, and Maharashtra) were studied because these were the initial foci of a large government-supported hypertension control program. Orally administered antihypertensive pills accounted for over 99.5% of overall sales (both in terms of value and volume); injectables, liquids, and powder forms were excluded from the analysis. The following indicators were analysed:

- Antihypertensive drug market in terms of overall volume (number of pills) and sales value (in local currency [INR]; calculated as price to retailer*drug sales volume).
- Sales by volume of antihypertensive drugs by class (i.e., calcium channel blockers [CCBs], beta-blockers [BBs], angiotensin II receptor blockers [ARBs], angiotensin converting enzyme inhibitors [ACEI], diuretics, alpha-blockers [AB], etc.) and drug molecule.

The Indian hypertension drug market grew at a compound annual growth rate (CAGR) of 6.9% from 2016 to 2018 with a total of 21,066 million antihypertensive pill sales in 2018 at a value of INR 87.36 billion (USD 1.28 billion). Of the five selected states, Maharashtra had the highest sales, while Punjab had the fewest. However, Punjab had the highest growth of antihypertensive sales during the study period, whereas Kerala witnessed the lowest (Table 1).

**Table 1:** Antihypertensive drugs sales volumes and value for India and five selected states 2016–2018.

Name of State	Sales volume (pills in million)				Sales value (INR million)			

	2016	2017	2018	CAGR*	2016	2017	2018	CAGR*

Maharashtra	3018	3237	3486	7.5%	12,164	13,036	14,494	9.2%
Kerala	1614	1650	1710	2.9%	5818	5896	6290	4.0%
Telangana	747	747	800	3.5%	3199	3299	3624	6.4%
Madhya Pradesh	661	704	765	7.6%	2762	2956	3304	9.4%
Punjab	505	589	693	17.1%	2146	2483	3003	18.6%
**All India**	**18,429**	**19,555**	**21,066**	**6.9%**	**74,360**	**78,915**	**87,357**	**8.4%**

* CAGR: compound annual growth rate.

There were 182 different antihypertensive drugs sold in India in 2018 of which 134 were fixed-dose, single-pill combinations (SPCs). Two-drug SPCs had the largest variety with 105 different combinations sold under more than 1000 brands by several manufacturers.

The sales of SPCs contributed to 39.1% of the total antihypertensive market by volume in 2018. Two-drug SPCs had the highest market share (36% of market volume), followed by CCBs (18%), BBs (16%), and ARBs (14%). While shares of most drug classes have increased since 2016, only ACEI exhibited a downward growth rate (–1.9%). Three-or-more-drug SPCs have grown at the highest rate since 2016, at 16.1%, followed by ARBs (11.1%) and CCBs (9.3%) (Figure 1).

**Figure 1:**
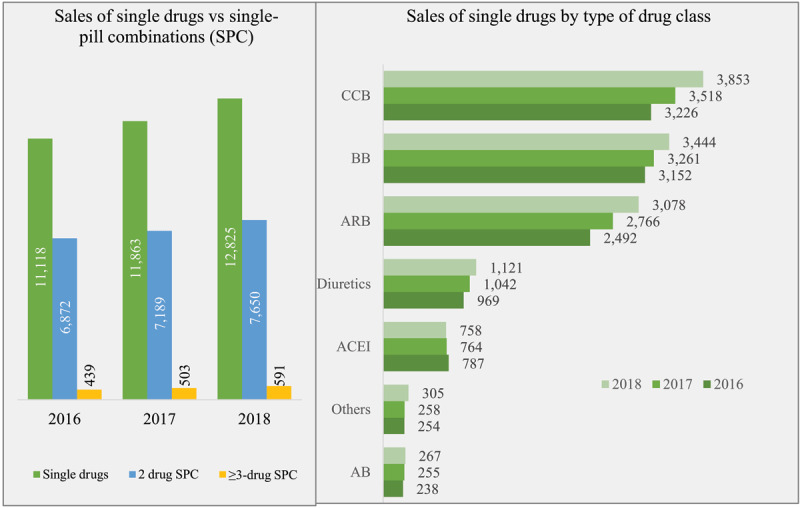
Antihypertensive drugs market in India: Total volume of sales (pills in millions) by type of formulation and drug class in 2016–2018. *Note*: “Others” includes several different categories of antihypertensive drugs, including clonidine, methyl dopa, moxonidine, dihydralazine, and aliskerin, etc.

Twenty-five antihypertensive drugs accounted for 80% of total volume of sales in 2018. From 2016–2018, the list of these 25 drugs has remained constant, except for a three-drug SPC (amlodipine-hydrochlorothiazide-telmisartan) that entered this list in 2018 replacing a two-drug SPC (furosemide-spironolactone). Amlodipine, amlodipine-atenolol, and telmisartan were the top three preferred drugs and their sales volumes contributed to 11%, 10%, and 9% of total sales in the country in 2018 respectively, followed by metoprolol succinate (6%), telmisartan-hydrochlorothiazide (4%), and amlodipine-telmisartan (4%). Atenolol dropped in its ranking from fifth place in 2016 to seventh place in 2018 due to decreasing sales volume.

There were inter-state variations in the market share of antihypertensive drug classes. In four states (except Kerala), two-drug SPCs had the highest market share and accounted for approximately 40% of all antihypertensives sold (range 38%–42%). BBs was the second most sold drug class in Punjab (19%) and Telangana (18%), while CCBs were preferred in Maharashtra (18%) and Madhya Pradesh (16%). In Kerala, CCBs (25%) had maximum sales, followed closely by BBs (21%) and two-drug SPCs (20%).

Procurement of drugs in India’s public sector varies from state to state, and product selection is usually based on the national or state essential drug list. Amlodipine, atenolol, losartan, and enalapril were the commonly procured antihypertensive drugs in the public sector, but their volume of procurement is not available in the public domain.

Though the private antihypertensive drugs market seems massive, another study based on the same dataset showed that there is a significant unmet need for hypertension treatment with only 21.5% of the estimated hypertensive adults in the country receiving drug treatment from the private market [2].

Recent national [3] and international guidelines [4] recommend CCBs, ARBs, ACEI, or diuretics for first-line treatment of uncomplicated hypertension. The Indian market seems aligned with these guidelines, as CCBs and ARBs were among the top sold drug classes and had higher growth rate compared to other drug classes. Even though first- and second-generation BBs have been shown to be inferior to other antihypertensive drugs for primary prevention of major cardiovascular events and stroke in typical patients [5,6], BBs, either singly or in combination with other antihypertensive drug classes, had a large market share, suggesting many prescribers still use BBs for treatment of uncomplicated hypertension. Significant state-to-state differences in sales volumes of different antihypertensive drug classes indicated variable treatment practices in the private sector in India, underscoring the need for private sector engagement strategies for cultivating standardized, evidence-based practice. Dissemination and uptake of standardised hypertension guidelines in the public and private sector can improve the quality of hypertension care and help consolidate the market, leading to lower prices of recommended drugs.

As a high proportion of people with hypertension require two or more drugs to achieve blood pressure control, antihypertensive drug SPCs provide benefits to patients due to decreased pill burden, improved treatment adherence and greater blood pressure reduction with fewer side-effects [7]. Despite the fact that antihypertensive drugs SPCs are not included in India’s current national essential medicines list (EML) [8], they contributed to almost two-fifths of the total antihypertensive drugs market over the study period, highlighting their popularity among the private health sector. In July 2019, the World Health Organization (WHO) included SPCs for hypertension management in its EML [9]. The prices of SPCs under the Government of India’s flagship generic drug scheme are lower than prices of the individual components [10]. In addition, a recent study showed that prices of SPCs in India’s private sector are not essentially higher than individual components [11]. Based on superior efficacy, treatment efficiency popularity among prescribers, availability and potential cost-effectiveness of SPCs, India may wish to consider inclusion of select antihypertensive SPCs in the national EML to extend their benefits to the public health system. Experiences from other parts of the world, such as the HEARTS in the Americas program in Latin America and the Caribbean, have demonstrated the acceptance and feasibility of standardized antihypertensive treatment protocols with the use of two-medication combination therapy in the initial treatment of newly diagnosed individuals with hypertension [12,13].
